# Orally Administrated Ascorbic Acid Suppresses Neuronal Damage and Modifies Expression of SVCT2 and GLUT1 in the Brain of Diabetic Rats with Cerebral Ischemia-Reperfusion

**DOI:** 10.3390/nu6041554

**Published:** 2014-04-15

**Authors:** Naohiro Iwata, Mari Okazaki, Meiyan Xuan, Shinya Kamiuchi, Hirokazu Matsuzaki, Yasuhide Hibino

**Affiliations:** 1 Laboratory of Immunobiochemistry, Faculty of Pharmaceutical Sciences, Josai University, Saitama 350-0295, Japan; E-Mails: n-iwata@josai.ac.jp (N.I.); genbien@josai.ac.jp (M.X.); kamiuchi@josai.ac.jp (S.K.); seitaib@josai.ac.jp (Y.H.); 2 Laboratory of Pharmacology, Faculty of Pharmaceutical Sciences, Josai University, Saitama 350-0295, Japan; E-Mail: ma-tsu@josai.ac.jp

**Keywords:** ascorbic acid, diabetes mellitus, oxidative stress, apoptosis, proinflammatory, cytokine, sodium-dependent vitamin C transporter 2 (SVCT2), glucose transporter 1 (GLUT1), rat, streptozotocin, middle cerebral artery occlusion and reperfusion

## Abstract

Diabetes mellitus is known to exacerbate cerebral ischemic injury. In the present study, we investigated antiapoptotic and anti-inflammatory effects of oral supplementation of ascorbic acid (AA) on cerebral injury caused by middle cerebral artery occlusion and reperfusion (MCAO/Re) in rats with streptozotocin-induced diabetes. We also evaluated the effects of AA on expression of sodium-dependent vitamin C transporter 2 (SVCT2) and glucose transporter 1 (GLUT1) after MCAO/Re in the brain. The diabetic state markedly aggravated MCAO/Re-induced cerebral damage, as assessed by infarct volume and edema. Pretreatment with AA (100 mg/kg, p.o.) for two weeks significantly suppressed the exacerbation of damage in the brain of diabetic rats. AA also suppressed the production of superoxide radical, activation of caspase-3, and expression of proinflammatory cytokines (tumor necrosis factor-α and interleukin-1β) in the ischemic penumbra. Immunohistochemical staining revealed that expression of SVCT2 was upregulated primarily in neurons and capillary endothelial cells after MCAO/Re in the nondiabetic cortex, accompanied by an increase in total AA (AA + dehydroascorbic acid) in the tissue, and that these responses were suppressed in the diabetic rats. AA supplementation to the diabetic rats restored these responses to the levels of the nondiabetic rats. Furthermore, AA markedly upregulated the basal expression of GLUT1 in endothelial cells of nondiabetic and diabetic cortex, which did not affect total AA levels in the cortex. These results suggest that daily intake of AA attenuates the exacerbation of cerebral ischemic injury in a diabetic state, which may be attributed to anti-apoptotic and anti-inflammatory effects via the improvement of augmented oxidative stress in the brain. AA supplementation may protect endothelial function against the exacerbated ischemic oxidative injury in the diabetic state and improve AA transport through SVCT2 in the cortex.

## 1. Introduction

Diabetes mellitus is a metabolic disorder associated with chronic hyperglycemia, which is known to enhance systemic oxidative stress, predisposing to diabetic complications. Diabetes is a major risk factor for atherosclerotic diseases such as acute brain ischemia [1,2]. Moreover, it increases the risks of morbidity and mortality after stroke [3,4]. Oxidative stress plays an essential role in the pathogenesis of transient cerebral ischemic injury [3,4,5]. In particular, reperfusion after a long period of vessel occlusion triggers the explosive generation of reactive oxygen species (ROS), such as superoxide radical (O_2_^–^), hydroxyl radical, hydrogen peroxide, *etc*., which causes apoptosis and delayed death of cells through oxidative damage to lipids, proteins, and DNA in the ischemic penumbral region [6,7,8,9]. In addition to apoptotic cell death, inflammatory neurodegeneration is another crucial process contributing to cerebral damage after ischemia and reperfusion [10]. ROS have been shown to activate nuclear factor-κB, which enhances the transcription of the genes encoding proinflammatory cytokines such as tumor necrosis factor-α (TNF-α) and interleukin-1β (IL-1β), leading to inflammatory responses [11]. Myeloperoxidase (MPO) expressed by microglia, a histopathological marker of inflammation, generates cytotoxic ROS and leads to further inflammatory damage in the ischemic tissue [12]. Accumulating evidence indicates that hyperglycemia in diabetes is associated with a decrease in the antioxidant potential and an increase in ROS generation [13,14,15]. In addition, diabetes has been shown to be a proinflammatory state that increases the risk of vascular complications [16,17]. Thus, the enhanced oxidative stress and inflammatory responses in the diabetic state may substantially contribute to the aggravation of cerebral injury caused by transient ischemia and subsequent reperfusion.

l-ascorbic acid (AA) is an essential antioxidant for scavenging free radicals in the brain. AA participates not only in sustaining the normal function of the central nervous system (CNS) but in ameliorating the damage induced by pathological conditions that increase the generation of ROS [18]. The CNS maintains relatively high concentrations of AA, indicating a neuroprotective role for AA [19]. The transport of AA from the plasma to the CNS is mainly mediated by sodium-dependent vitamin C transporter 2 (SVCT2). In addition, glucose transporter 1 (GLUT1) is located in the endothelial cells of the blood–brain barrier (BBB) and transports oxidized AA ([dehydroascorbic acid (DHA)) as another source of AA to the brain. Upregulation of SVCT2 [20] and GLUT1 [21] expression has been demonstrated in rats with cerebral ischemic injury, which suggests that AA is necessary for protection against oxidative neuronal injury. The concentration of AA is considered to reflect oxidative stress in animal tissues sensitively [22]. Patients with ischemic stroke or diabetes have lower concentrations of AA in the plasma, suggesting that a systemic decrease in AA is a consequence of enhanced consumption of AA by elevated oxidative stress [23,24]. In a previous study, we demonstrated that chronic supplementation with AA attenuates oxidative stress in both the plasma and the brain and alleviates cerebral injury induced by middle cerebral artery occlusion and reperfusion (MCAO/Re) in rats with streptozotocin (STZ)-induced diabetes [25]. We showed that the activity of antioxidant enzymes (superoxide dismutase, catalase, and glutathione peroxidase) is decreased, and lipid peroxidation is accelerated in the brain of diabetic rats; those detrimental oxidative processes are inhibited by AA supplementation. These data suggest that the enhanced oxidative stress in the diabetic state causes the functional impairment of antioxidant enzymes, and the resulting diminution in antioxidative defense can cause further enhancement of the generation of ROS and subsequent neuronal apoptosis and inflammatory neurodegeneration in the ischemic brain. Nonetheless, there is a shortage of studies so far on the antiapoptotic and anti-inflammatory effects of AA in cerebral ischemia with diabetes.

In the present study, we evaluated the effects of chronic oral pretreatment with AA on the production of O_2_^–^ and on apoptosis in the brain after MCAO/Re in rats with STZ-induced diabetes. To test whether AA suppresses inflammatory responses induced by MCAO/Re, we also examined the effects of AA on the expression of TNF-α, IL-1β, and MPO in the brain. Although the transport of AA to the CNS is an important factor for its neuroprotection, changes in expression of SVCT2 and GLUT1 proteins in response to AA supplementation and/or cerebral ischemia in diabetic state have not been reported. Therefore, we investigated the effects of AA supplementation on the expression of SVCT2 and GLUT1 after MCAO/Re in the brain of diabetic rats.

## 2. Experimental Section

### 2.1. Experimental Animals

Animal care and surgical procedures were performed in accordance with guidelines approved by the National Institutes of Health (Bethesda, MD, USA) and the Josai University Animal Research Committee. Male Sprague-Dawley rats (4 weeks old, weight 120–140 g) were purchased from Japan SLC (Shizuoka, Japan) and were housed under standard conditions with a temperature-controlled environment (23 °C ± 0.5 °C) and a 12 h light/dark cycle. The animals were allowed free access to rodent chow (CE-2, CLEA Japan, Tokyo, Japan) and water. Type 1 diabetes was induced in the rats (diabetic group) by a single intraperitoneal injection of STZ (50 mg/kg of body weight) dissolved in 0.1 mM sodium citrate, pH 4.5, while the normal control rats (nondiabetic group) were injected with the buffer only [26]. Seven days after the injection of STZ, a blood sample was collected by tail vein paracentesis, following which plasma glucose was measured using a glucose analyzer (Ascensia, Bayer Yakuhin, Osaka, Japan). Diabetes was defined as a blood glucose level greater than 300 mg/dL. Then, the diabetic and nondiabetic groups were divided into 2 groups and were housed for an additional 6 weeks until stroke was induced by MCAO/Re. AA (l-Ascorbic acid, Wako Pure Chemicals Industries, Osaka, Japan) (100 mg/kg; nondiabetic and diabetic AA-supplemented groups) or distilled water (nondiabetic and diabetic control groups) was orally administered through nasogastric tube once daily for the last 2 weeks. AA was stored at +4 °C, and dissolved in distilled water fresh each time just before administration.

### 2.2. MCAO/Re

The experimental MCAO/Re rat model was prepared as described previously [25]. The rats were anesthetized with halothane (4% for induction and 1.5% for maintenance) under spontaneous respiration. After a midline incision on the neck, the right common carotid artery was isolated under an operating microscope. All branches of the external carotid artery were ligated. The tip of a 4–0 surgical nylon monofilament rounded by flame heating was inserted through the internal carotid artery and advanced to occlude the origin of MCA. The rectal temperature was maintained at 37 °C with a heat lamp and a heating pad during the operation. After 2 h of occlusion, the filament was withdrawn to enable reperfusion. The distance from bifurcation of the common carotid artery to the tip of the suture was approximately 20 mm in all rats. Cerebral blood flow was detected using a Laser Doppler flowmetry (ATBF-LC1, Unique Medical, Tokyo, Japan), and approximately 50% reduction of its baseline associated with MCAO was ascertained in the rats. Then, the rats were allowed to recover from anesthesia at room temperature and were killed after 24 h of reperfusion. Sham operation involved the same manipulations but insertion of the filament.

### 2.3. Infarct and Edema Assessment

After 24 h of reperfusion, the rats were subjected to general halothane anesthesia and decapitated. The brain was immediately removed and placed in ice-cold saline. Each brain was then cut into 2 mm coronal slices in a rat brain matrix. The brain slices were immediately immersed in 2% 2,3,5-triphenyl tetrazolium chloride (TTC, Wako Pure Chemicals Industries, Osaka, Japan) at 37 °C for 15 min and then in 4% formaldehyde [26,27]. Infarct areas were identified using an image analysis system (Scion Image 1.62, Frederick, MD, USA) and were combined to obtain the infarct volumes per brain according to the following formula: corrected infarct volume (%) = (left hemisphere volume − (right hemisphere volume − the infarct volume)) × 100/left hemisphere volume. Edema in the ischemic hemisphere was also calculated as follows: edema (%) = (right hemisphere volume − the infarct volume)/left hemisphere volume × 100.

### 2.4. Neurological Evaluation

Postischemic neurological deficits were evaluated after 24 h of reperfusion on a 5-point scale as follows: grade 0, no deficit; grade 1, failure to fully extend the right forepaw; grade 2, spontaneous circling or walking to a contralateral side; grade 3, walking only when stimulated; grade 4, unresponsive to stimulation and a depressed level of consciousness; and grade 5, death [25,27]. Before MCAO, the neurological score was zero in all the rats. The rats that did not exhibit neurological deficits after MCAO/Re were excluded from the study. Grades of the neurological score were evaluated by an investigator blinded to the treatment protocol.

### 2.5. Detection of O_2_^−^ Production in the Brain

Detection of intracellular O_2_^−^ production in the ischemic penumbral region of the cortex after MCAO/Re was performed by histochemical staining of freshly frozen brain sections (8 μm thick) with the fluorescent probe dihydroethidium (DHE). The brain sections were immediately incubated with DHE (10 μmol/L, Sigma-Aldrich Japan, Tokyo, Japan) in phosphate-buffered saline for 30 min at 37 °C [28,29]. To determine the fluorescence intensity of oxidized DHE, 3 visual fields within the penumbral cortex regions of each hemisphere were photographed using a confocal laser scanning microscope (Fluoview FV1000, OLYMPUS, Tokyo, Japan) with excitation at 510 nm and emission at 580 nm. Fluorescence intensity of oxidized DHE was quantified using imaging software (FV10-ASW 1.7, OLYMPUS, Tokyo, Japan). Analyses of immunohistochemistry were performed by an investigator blinded to the treatment protocol.

### 2.6. Immunohistochemistry

Immunohistochemical staining was performed as described previously [20,30,31]. The brain was fixed with 4% phosphate-buffered paraformaldehyde. Coronal brain sections (8 μm thick) were incubated with 3% hydrogen peroxide for 40 min at room temperature to inhibit endogenous peroxidase and then incubated with blocking buffer (4% Block Ace, Dainippon Sumitomo Pharma, Osaka, Japan) for 2 h. Then, the slices were incubated with polyclonal rabbit anti-IL-1β antibody (1:300, Santa Cruz Biotechnology, Dallas, TX, USA), polyclonal rabbit anti-TNF-α antibody (1:200, Rabbit mAb, Hycult Biotech, Uden, The Netherlands), polyclonal rabbit anti-cleaved caspase-3 antibody (1:100, Cell Signaling Technology, Danvers, MA, USA), or monoclonal mouse anti-MPO (1:100, Hycult Biotech, Uden, The Netherlands), polyclonal rabbit anti-SVCT2 antibody (1:100, Santa Cruz Biotechnology, Dallas, TX, USA), polyclonal rabbit anti-GLUT1 antibody (1:100, Santa Cruz Biotechnology, Dallas, TX, USA) in 0.01 mol/L phosphate-buffered saline overnight at 4 °C. In a double-immunohistchemical study for determination of the cell types of expressing SVCT2 or GLUT1, monoclonal mouse anti-neuronal nuclei (NeuN) antibody (1:300, Millipore, Billerica, MA, USA) and monoclonal mouse anti-rat endothelial cell antigen (RECA1) antibody (1:200, Abcom, Cambridge, UK) were used. After washing with phosphate-buffered saline, the slices were incubated with either Cy3- or FITC-conjugated secondary antibody (1:200, Millipore, Billerica, MA, USA) for 2 h at room temperature. Finally, the sections were incubated with the nuclear stain TO-PRO-3 (1:10,000, Invitrogen, Carlsbad, CA, USA) in phosphate-buffered saline for 10 min at room temperature with gentle agitation. Immunofluorescence was visualized using the laser scanning confocal microscope and the intensity was measured using the imaging software. Three sections per rat and 3–4 rats per group were used for the analyses.

### 2.7. Real-Time Polymerase Chain Reaction (PCR) Analysis

The expression levels of SVCT2 (*Slc23a2*) and GLUT1 (*Slc2a1*) mRNA were assessed by quantitative real-time polymerase chain reaction (PCR) as described previously [32]. The rats subjected to MCAO were killed after 24 h of reperfusion, and total RNA samples were prepared from the ischemic penumbral cortex of each rat. Total RNA was extracted using the RNeasy Mini Kit (QIAGEN, Hilden, Germany) according to the manufacturer’s protocol. Total RNA (500 pg) from each sample was reverse-transcribed with oligo-dT and random hexamer primers using reverse transcriptase (PrimeScript RT Enzyme Mix I; Takara RNA PCR Kit, Takara Biomedicals, Shiga, Japan). Real-time PCR was performed with 10 ng of cDNA and a pair of gene-specific primers (Takara Biomedicals, Shiga, Japan) that were added to the SYBR Premix EX *Taq* (Takara Biomedicals, Shiga, Japan) and subjected to PCR amplification on the iCycler iQ Real-Time Detection System (Bio-Rad Laboratories, Hercules, CA, USA) (1 cycle at 95 °C for 10 s and 50 cycles at 95 °C for 5 s and 60 °C for 34 s). β-Actin expression was used to normalize the cDNA levels. The PCR products were analyzed using a melting curve to ascertain specificity of the amplification. The data were expressed as mean ± SD relative to the sham-operated nondiabetic group.

### 2.8. Measurement of Total AA Level

Total AA (AA + DHA) levels in the plasma and cortex have been determined by spectrophotometric method using Vitamin C Assay kit (ROIK02, Shima Laboratories, Tokyo, Japan) which is based on 2,4-dinitrophenylhydrazine method [33]. Briefly, plasma was mixed with an equal volume of 10% metaphosphoric acid solution. Cortex tissue was mixed with 14 times volume of 5.4% metaphosphoric acid solution and homogenized. After centrifugation of the solutions at 10,000× *g* for 15 min at 4 °C, the supernatants were used to the assay.

### 2.9. Statistical Analysis

Two-way ANOVA, followed by *post hoc* Tukey’s multiple-comparison test, was used for statistical analysis. Neurological deficit scores were analyzed using the Kruskal-Wallis test, followed by the Mann-Whitney *U* test. In all cases, a *p* value of <0.05 was assumed to denote statistical significance.

## 3. Results

### 3.1. Blood Glucose and Body Weight

Body weight and blood glucose data from the experimental rats were obtained throughout the study period (Table 1). Similar to our previous study [25], the diabetic control group of rats had a significantly decreased body weight and an increased blood glucose level compared with the nondiabetic control group. There were no significant differences in those parameters between the AA-supplemented groups and their controls.

**Table 1:** Effects of oral supplementation with AA on body weight and blood glucose levels in nondiabetic (non-DM) and diabetic (DM) groups of rats.

Groups	Body Weight (g)	Blood Glucose (mg/dL)
Non-DM	335 ± 16	112 ± 11
Non-DM + AA	340 ± 23	135 ± 9
DM	255 ± 32 *	485 ± 42 *
DM + AA	269 ± 36 *	469 ± 71 *

The data are shown as mean ± SD. * *p* < 0.01 *vs*. the nondiabetic group (*n =* 6–7).

### 3.2. Ischemic Brain Injury and Neurological Deficits

**Figure 1:**
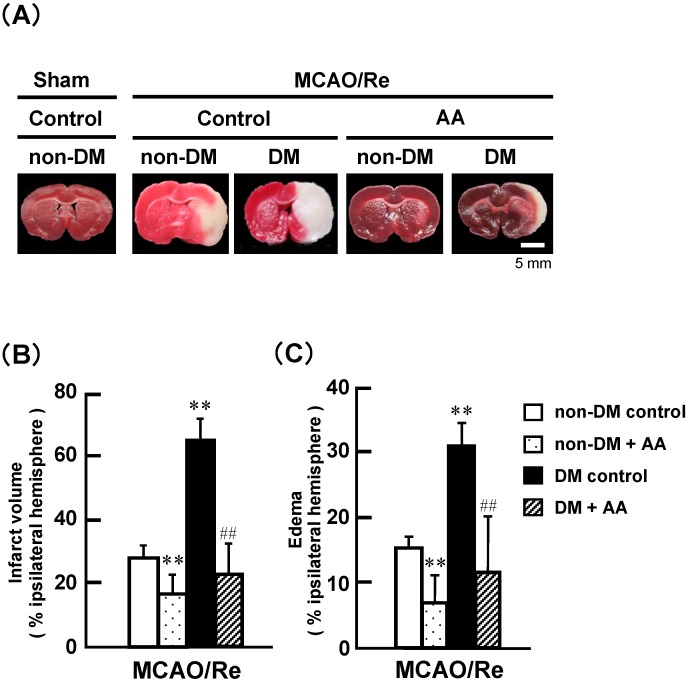
Effects of AA supplementation on infarction induced by MCAO/Re in the brain of nondiabetic and diabetic rats. (**A**) Representative photographs of staining of coronal brain sections from the rats of the nondiabetic + sham operation group and from distilled water-administered (Control) or AA (100 mg/kg)-supplemented nondiabetic or diabetic groups with MCAO with reperfusion (MCAO/Re). AA or water was orally administered once daily for 2 weeks; (**B**) Infarct volume in ischemic hemispheres of the diabetic and nondiabetic groups after MCAO/Re by TTC staining; (**C**) Edema volume in ischemic hemispheres of the diabetic and nondiabetic groups after MCAO/Re. Data are presented as means ± SD (*n* = 6–7). ** *p* < 0.01 compared with the nondiabetic control group. ^##^ *p* < 0.01 compared with the diabetic control group. DM in the figure denotes diabetic, while non-DM denotes nondiabetic.

Figure 1 shows MCAO/Re-induced brain injury in the nondiabetic control, nondiabetic + AA, diabetic control, and diabetic + AA groups of rats. Examples of TTC staining in the coronal brain sections at 24 h after MCAO/Re are shown in Figure 1A. The infarct developed in the corpus striatum and cortex of the nondiabetic control rats. In the diabetic rats, the infarction zone was remarkably enlarged and extended to a large part of the left striatum and cortex. In contrast, the infarcts in the AA-supplemented groups were smaller than those in their respective controls. Quantitative assays revealed that the infarct volume and edema in the diabetic control group were significantly increased by approximately 2.5-fold and 2-fold, respectively, compared with those in the nondiabetic control group (Figure 1B,C). AA supplementation in the nondiabetic group significantly decreased infarction and edema. Furthermore, AA almost completely suppressed the exacerbation of brain damage by diabetes.

**Figure 2:**
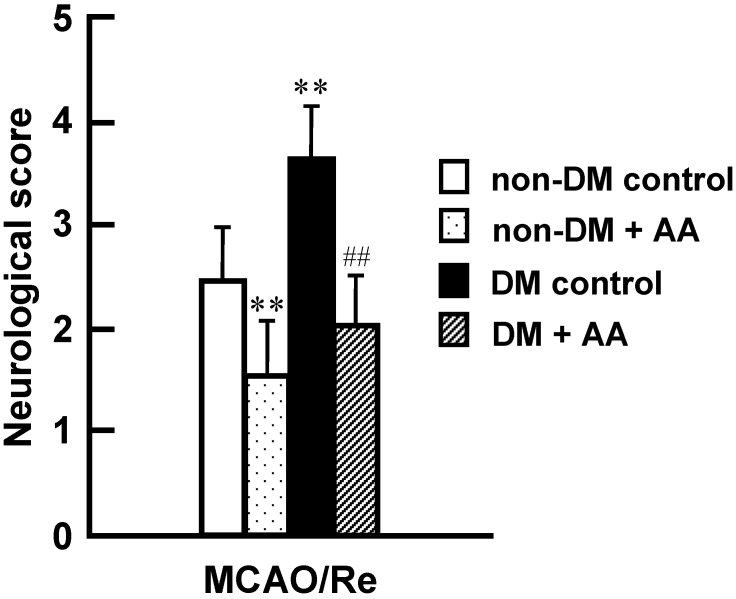
Effects of AA supplementation on neurological deficits induced by MCAO/Re in nondiabetic and diabetic rats. Postischemic neurological deficits were evaluated on a 5-point scale at 24 h of reperfusion after 2 h of MCAO. Data are mean ± SD of 6–7 rats per group. ** *p* < 0.01 compared with the nondiabetic control group. ^##^ *p* < 0.01 compared with the diabetic control group. DM in the figure denotes diabetic, while non-DM denotes nondiabetic.

Consistent with the aforementioned results of the brain injury experiments, neurological deficits were exacerbated in the diabetic control group of rats (Figure 2). Compared with the diabetic control group, the diabetic + AA group showed significant alleviation of the neurological deficits.

### 3.3. O_2_^–^ Production after Ischemia with Reperfusion

**Figure 3:**
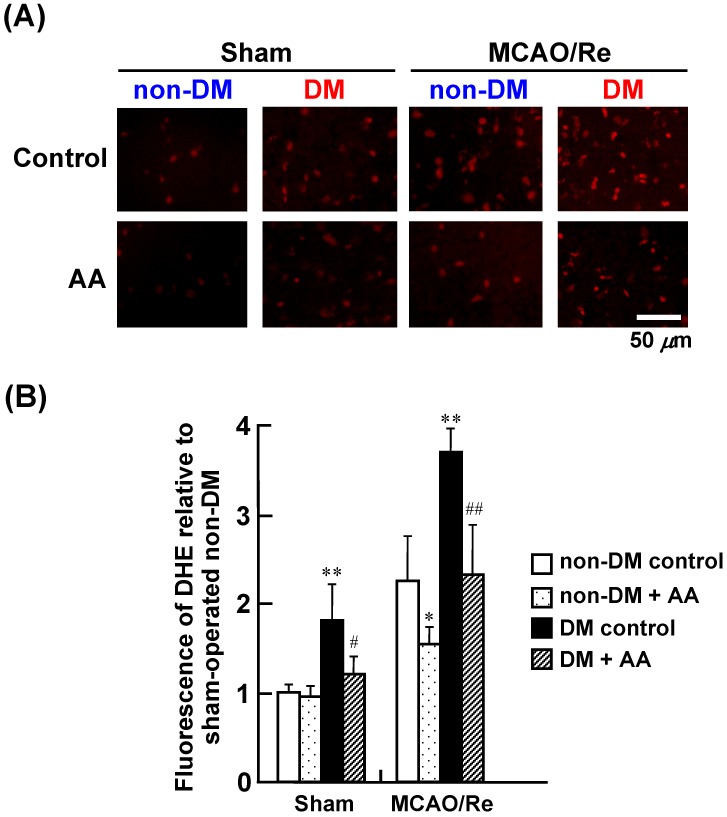
Effects of AA supplementation on production of O_2_^–^ after MCAO/Re in the brain of nondiabetic and diabetic rats. (**A**) Representative photographs of superoxide production detected by DHE staining in coronal sections of the cortex from the nondiabetic and diabetic rats; (**B**) Quantitative analysis of DHE fluorescence intensity in the cortex. The data are presented as mean ± SD (*n* = 3–4). * *p* < 0.05, ** *p* < 0.01 compared with the nondiabetic control group. ^#^ *p* < 0.05, ^##^ *p* < 0.01 compared with the diabetic control group. DM in the figure denotes diabetic, while non-DM denotes nondiabetic.

Figure 3 shows fluorescence intensity of DHE in the penumbral cortex, which depends on intracellular O_2_^–^ production. Representative histological images of DHE staining in the nondiabetic control, nondiabetic + AA, diabetic control, and diabetic + AA groups are shown in Figure 3A. DHE-positive cells with low fluorescence intensity were sparsely distributed in the cortex of sham-operated nondiabetic rats. In contrast, sham-operated diabetic rats had an increased number of DHE-positive cells with higher fluorescence intensity in the cortex, indicating basal augmentation of the generation of ROS in the brain of the diabetic rats. The intensity of DHE fluorescence was remarkably increased by MCAO/Re in the nondiabetic rats and was further augmented in the diabetic rats, suggesting that the exacerbated injury can be attributed to enhanced generation of ROS in diabetes. AA supplementation significantly reduced the fluorescence of DHE in the cortex of the nondiabetic and diabetic rats compared with that in their respective controls.

### 3.4. Apoptosis Induced by Ischemia with Reperfusion

**Figure 4:**
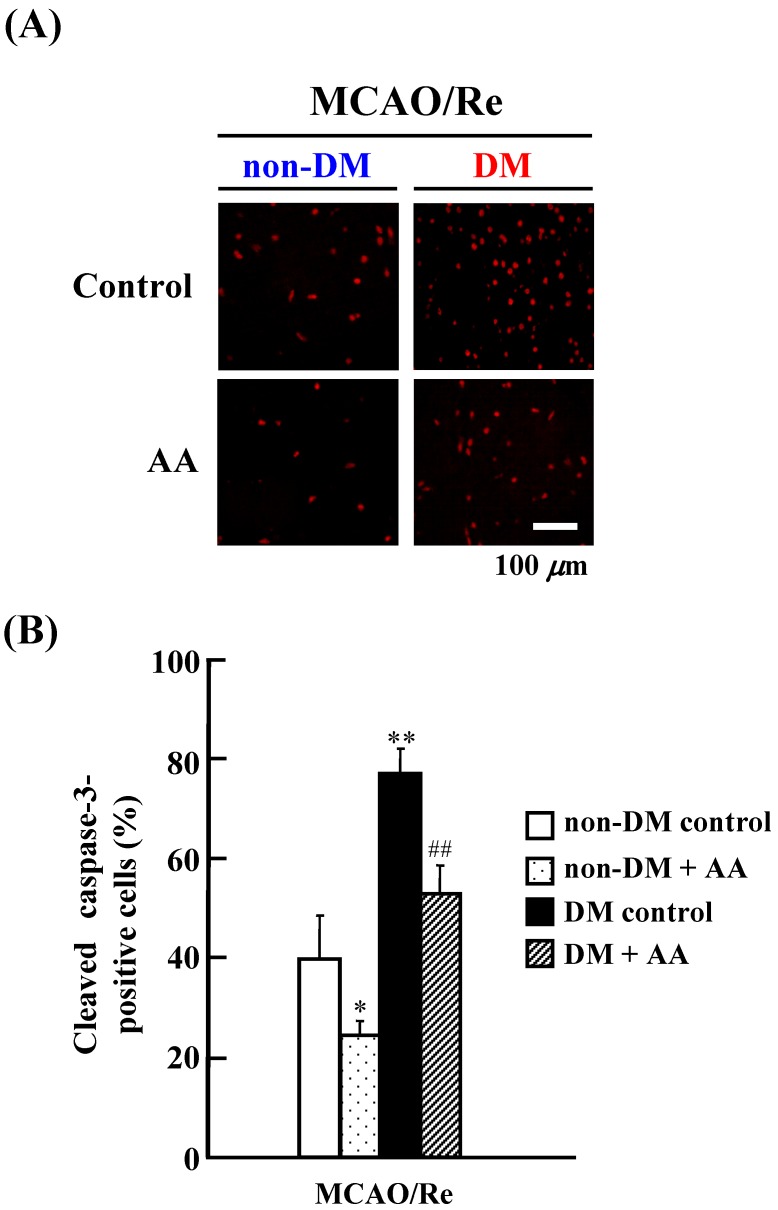
Effects of AA supplementation on cleaved caspase-3 after MCAO/Re in the brain of nondiabetic and diabetic rats. (**A**) Representative photographs of cleaved caspase-3 immunostaining in the cortex coronal sections of nondiabetic and diabetic rats; (**B**) Quantitative analysis of cleaved caspase-3 positive cells (fluorescence intensity in the cortex). The data are presented as mean ± SD (*n* = 3–4). * *p* < 0.05, ** *p* < 0.01 compared with the nondiabetic control group. ^##^ *p* < 0.01 compared with the diabetic control group. DM in the figure denotes diabetic, while non-DM denotes nondiabetic.

Activation of caspase-3, a key mediator of the execution phase of apoptosis, was determined by immunostaining for cleaved caspase-3, which is an activated form of this enzyme, in the ischemic penumbral cortex of the four experimental groups after MCAO/Re (Figure 4). Compared with the nondiabetic control group, the number of cleaved caspase-3 positive cells was remarkably increased by MCAO/Re in the diabetic control group. AA significantly attenuated the MCAO/Re-induced activation of caspase-3 in the nondiabetic and diabetic groups.

### 3.5. Expression of IL-1β, TNF-α, and MPO in the Cortex

**Figure 5:**
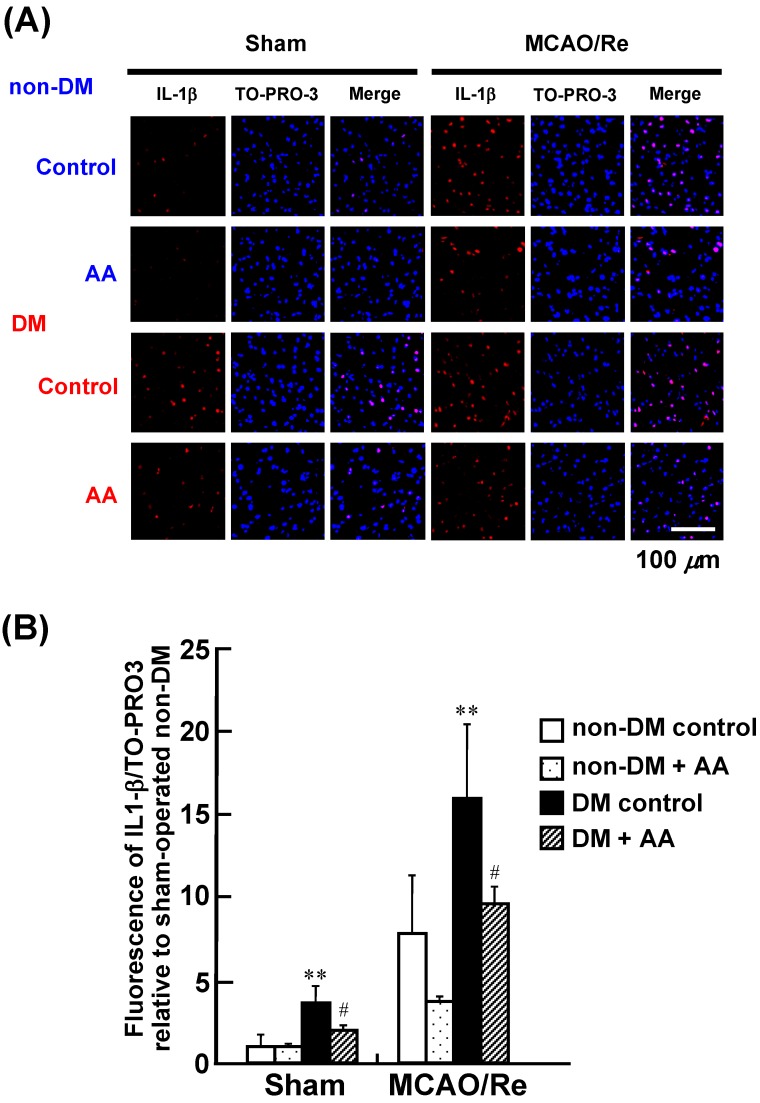
Effects of AA supplementation on expression of IL-1β in the penumbral cortex after MCAO/Re in the brain of nondiabetic and diabetic rats. (**A**) Representative photographs of IL-1β immunostaining (red fluorescence) and staining of nuclei by TO-PRO-3 (blue fluorescence) in the cortex coronal sections of nondiabetic and diabetic rats; (**B**) Quantitative analysis of IL-1β fluorescence intensity in the cortex. The data are presented as mean ± SD (*n* = 3–4). ** *p* < 0.01 compared with the nondiabetic control group. ^#^ *p* < 0.05 compared with the diabetic control group. DM in the figure denotes diabetic, while non-DM denotes nondiabetic.

**Figure 6:**
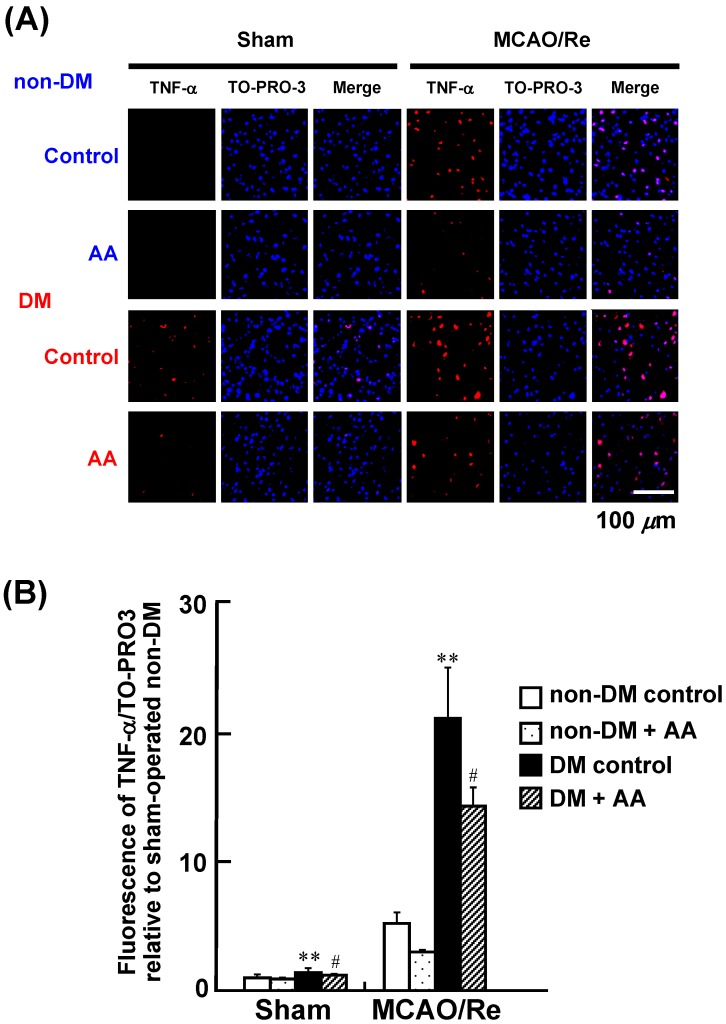
Effects of AA supplementation on expression of TNF-α in the penumbral cortex after MCAO/Re in the brain of nondiabetic and diabetic rats. (**A**) Representative photographs of TNF-α immunostaining (red fluorescence) and staining of nuclei by TOPRO-3 (blue fluorescence) in the cortex coronal sections of nondiabetic and diabetic rats; (**B**) Quantitative analysis of TNF-α fluorescence intensity in the cortex. The data are presented as mean ± SD (*n* = 3–4). ** *p* < 0.01 compared with the nondiabetic control group. ^#^ *p* < 0.05 compared with the diabetic control group. DM in the figure denotes diabetic, while non-DM denotes nondiabetic.

To assess the effects of AA supplementation on the expression of proinflammatory cytokines, we performed immunohistochemical staining for IL-1β and TNF-α. This experiment confirmed upregulation of the protein level of these cytokines as a result of MCAO/Re and diabetes (Figure 5 and Figure 6). Quantification of the immunostaining data showed that the sham-operated diabetic control group had a significant increase in IL-1β and TNF-α expression compared with the sham-operated nondiabetic control group, suggesting basal augmentation of the inflammatory response in the diabetic brain. MCAO/Re significantly increased the expression levels of those proinflammatory cytokines in the nondiabetic cortex. The ischemia-induced upregulation of those cytokines was markedly accelerated by diabetes: the diabetic control group showed a 15.9- and 21.0-fold increase in IL-1β and TNF-α expression, respectively, compared with the nondiabetic control group. AA supplementation significantly suppressed the basal and ischemia-enhanced expression of these cytokines in diabetic rats.

**Figure 7:**
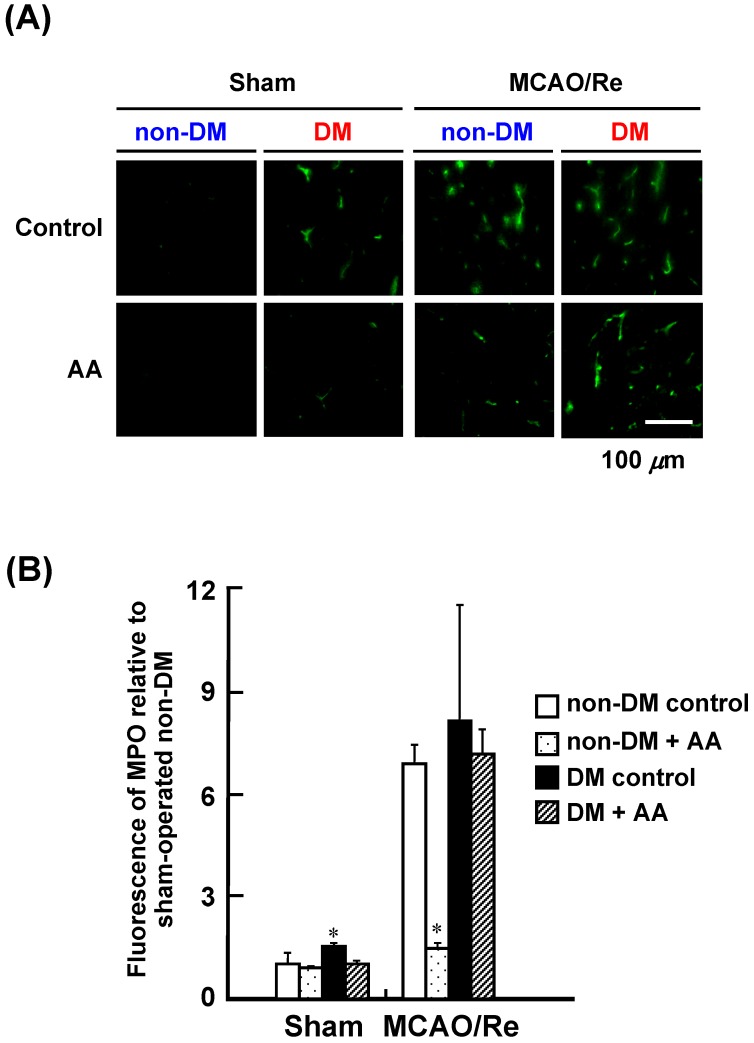
Effects of AA supplementation on MPO expression after MCAO/Re in the brain of nondiabetic and diabetic rats. (**A**) Representative photographs of MPO immunostaining in the cortex coronal sections of nondiabetic and diabetic rats; (**B**) Quantitative analysis of MPO fluorescence intensity in the cortex. The data are presented as mean ± SD (*n* = 3–4). * *p* < 0.05 compared with the nondiabetic control group. DM in the figure denotes diabetic, while non-DM denotes nondiabetic.

In the nondiabetic rat cortex, the protein expression of MPO was markedly upregulated after MCAO/Re (Figure 7). AA supplementation abrogated the ischemia-induced increase in MPO expression. Compared with the nondiabetic rats, the basal level of MPO expression increased only slightly in the diabetic rats. MCAO/Re upregulated the expression of MPO to the level similar to that in the nondiabetic group. AA supplementation had no effect on the ischemia-induced increase in the MPO level in the diabetic rats.

### 3.6. Expression of SVCT2 and GLUT1 in the Cortex

**Figure 8:**
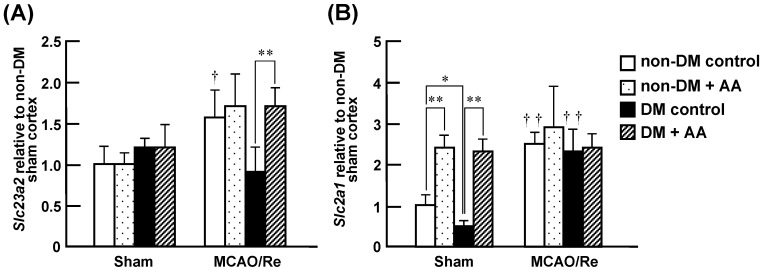
Effects of AA supplementation on SVCT2 and GLUT1 mRNA expression in the ischemic penumbral cortex of nondiabetic and diabetic rats. Expression levels of SVCT2 mRNA (*Slc23a2*) (**A**) and GLUT1 mRNA (*Slc2a1*) (**B**) were assessed using real-time PCR analysis of the penumbral cortex of nondiabetic and diabetic rats after MCAO/Re. The data are presented as mean ± SD (*n =* 3–6). **^†^***p* < 0.05, **^††^***p* < 0.01 compared with the respective sham-operated controls. * *p* < 0.05, ** *p* < 0.01.

The expression and transport activity of SVCT2 as a specific transporter of AA in the CNS have been reported to be enhanced after MCAO/Re in mice [20]. In addition, DHA is known to be transported across the BBB via GLUT1 [19]; thus, we investigated effects of AA supplementation on changes in the expression of SVCT2 and GLUT1 mRNA in diabetic animals with or without MCAO/Re (Figure 8). There was no significant difference in the basal expression levels of SVCT2 mRNA between the nondiabetic and diabetic groups or between each of them and their AA-supplemented groups. In the nondiabetic and nondiabetic + AA groups, the expression level of SVCT2 mRNA was equally upregulated at 24 h after MCAO/Re. In contrast, the upregulation of ischemia-induced SVCT2 mRNA expression was not observed in the diabetic control group, whereas it was observed in the diabetic + AA group. Compared with the nondiabetic control group, the diabetic state significantly downregulated the expression of GLUT1 mRNA in the cortex. AA supplementation increased the expression of GLUT1 in the nondiabetic and diabetic rats. In the nondiabetic and diabetic control groups, MCAO/Re had a stimulating effect on the expression of GLUT1 mRNA that was similar to the effect of AA supplementation. Further enhancement by ischemia was not detected in the AA-supplemented groups.

**Figure 9:**
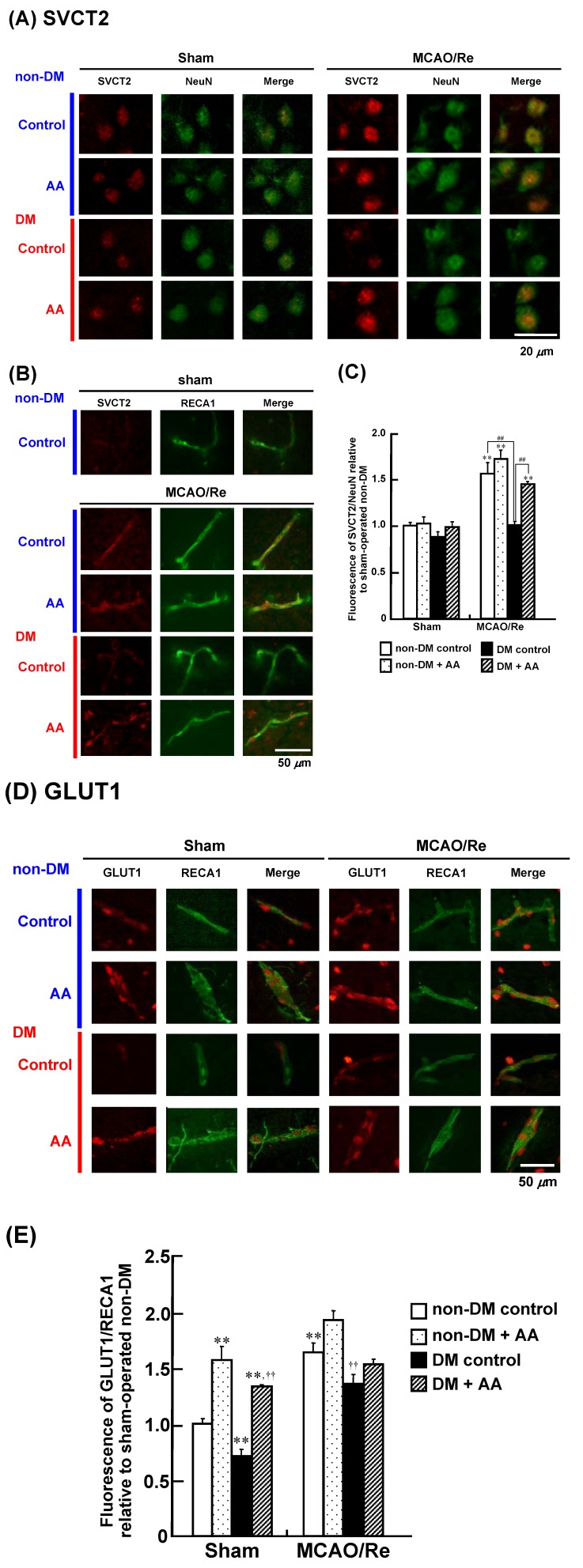
Effects of AA supplementation on SVCT2 and GLUT1 expression in the ischemic penumbral cortex of nondiabetic and diabetic rats. Expression levels of SVCT2 (**A**–**C**) and GLUT1 (**D**,**E**) were assessed using immunohistostaining of the penumbral cortex of nondiabetic and diabetic rats after MCAO/Re. Immunohistochemical expression of SVCT2 (red fluorescence) merged with NeuN (**A**) or with RECA1 (**B**) (green fluorescence); (**C**) Quantitative analysis of SVCT2 fluorescence intensity in the cortex. ^#^ *p* < 0.05, ^##^ *p* < 0.01. * *p* < 0.05, ** *p* < 0.01 compared with the respective sham-operated nondiabetic controls; (**D**) Immunohistochemical expression of GLUT1 (red fluorescence) merged with RECA1 (green fluorescence); (**E**) Quantitative analysis of SVCT2 fluorescence intensity in the cortex. * *p* < 0.05, ** *p* < 0.01 compared with the respective sham-operated nondiabetic controls. **^†^** *p* < 0.05, **^††^** *p* < 0.01 compared with the respective sham-operated diabetic controls.

To assess the effects of AA supplementation on the expression of SVCT2 protein after MCAO/Re in detail, the localization and the expression levels of SVCT2 in the penumbral cortex were examined by immunohistochemical staining (Figure 9A). Double immunofluorescence staining with antibodies of anti-SVCT2 and anti-NeuN, a biomarker for neurons, revealed that majority of the cells exhibiting SVCT2 expressed NeuN in the sham-operated nondiabetic and diabetic rat cortex (Figure 9A). In addition, the cells exhibiting the endothelial cell maker RECA1 showed a low level of SVCT2-immunoreactivity (Figure 9B). These expression levels in the neurons and capillary endothelial cells of SVCT2 were upregulated by MCAO/Re in the nondiabetic rats, but not in the diabetic rats. In agreement with the results from the mRNA measurement, quantification of the immunofluorescence revealed that the expression levels of SVCT2 immunoreactivity were significantly increased in response to MCAO/Re both in the nondiabetic control and AA-supplemented nondiabetic groups (Figure 9C). This reaction against MCAO/Re was abrogated in the diabetic control group, whereas the AA-supplemented diabetic rats showed an increased expression of SVCT2 in the cortex after MCAO/Re.

On the other hand, GLUT1 immunoreactivity was predominantly colocalized with RECA1 (Figure 9D). Some of the cells exhibiting NeuN showed a weak GLUT1-immunoreactivity (data not shown). In the sham-operated nondiabetic and diabetic groups with AA supplementation, the expression levels of GLUT1 in the capillary endothelial cells were remarkably upregulated. Additionally, MCAO/Re showed a stimulating effect on the GLUT1 expression both in the nondiabetic and diabetic cortex. Quantitative analyses of immunofluorescence in the sections revealed that the sham-operated diabetic control rat cortex had a lower expression level of GLUT1 compared with the nondiabetic rat cortex, and AA supplementation or MCAO/Re caused a significant increase in the expression of GLUT1 (Figure 9E).

### 3.7. Total AA Levels in the Plasma and Cortex

**Figure 10:**
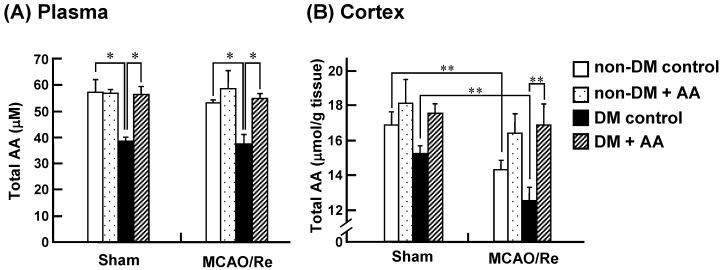
Effects of AA supplementation on levels of total AA (AA + DHA) in the plasma and cortex of nondiabetic and diabetic rats. Total AA (AA + DHA) levels in the plasma (**A**) and cortex (**B**) measured in nondiabetic and diabetic rats after MCAO/Re. The data are presented as mean ± SD (*n* = 3–6). * *p* < 0.05, ** *p* < 0.01.

To determine whether the changes in expression levels of SVCT2 and GLUT1 induced by AA supplementation or by MCAO/Re affect the concentrations of AA in the cortex, we measured total AA (AA + DHA) levels in the plasma and cortex of the experimental groups (Figure 10). It has been indicated that reduction of DHA to AA or decomposition of DHA to 2,3-diketo-1-gulonic acid is rapid in the plasma and brain, and that the concentrations of DHA in these tissues are 0%–2% of AA [19]. Therefore, total AA levels are considered to almost equal to the AA levels. The plasma of the sham-operated diabetic control rats had a significant smaller amount of total AA levels compared with the nondiabetic rats (Figure 10A). AA supplementation restored the decrement of the total AA levels in the diabetic rats to almost the same levels as those in the nondiabetic control rats. MCAO/Re had little effect on the total AA levels in the plasma of all groups. In contrast, neither diabetic state nor AA supplementation affected the total AA levels in the cortex of sham-operated groups. MCAO/Re significantly decreased the total AA levels both in the nondiabetic and diabetic cortex. AA supplementation to the diabetic rats caused a significant increase in the total AA levels in the cortex in response to MCAO/Re.

## 4. Discussion

The present study confirmed that the STZ-evoked diabetic state aggravates neuronal damage caused by a transient cerebral ischemia and subsequent reperfusion in rats. We found that diabetes enhances the production of O_2_^−^, activates caspase-3, and induces the expression of proinflammatory cytokines (TNF-α and IL-1β) in the brain. These detrimental effects are markedly potentiated by cerebral ischemia and reperfusion, leading to greater infarct growth and aggravation of apoptosis and inflammation. Our results show that chronic supplementation with AA inhibits the apoptotic changes and proinflammatory responses, and attenuates the exacerbation of cerebral injury and neurological deficits in the diabetic state. These beneficial effects of AA could be attributed to its antioxidant and anti-inflammatory properties. We observed that the expression level of SVCT2 increases in response to MCAO/Re in the nondiabetic cortex, which is accompanied by an increase in the total AA (AA + DHA) in the tissues, and that these responses are abolished in the diabetic rats. AA supplementation to the diabetic rats restored these responses to the levels of the nondiabetic rats. Therefore, supplementation with AA may enhance the transport of AA into neuronal cells, resulting in reinforcement of the antioxidant defense and alleviation of oxidative ischemic injury in the brain of diabetic rats.

Because rats can synthesize their own AA, unlike humans, they seem to hardly suffer from low levels of internal AA [19]. However, there is experimental evidence that STZ-induced type 1 diabetic rats have low levels of AA in the plasma, liver, kidney, and other tissues [23,34,35]. It is conceivable that the enhanced oxidative stress in diabetes consumes the circulating AA and that the resulting diminution in the AA concentration leads to further enhancement of the reactions mediated by free radicals. This study indicates that the diabetic rats have low levels of AA in the plasma, and that chronic oral pretreatment with AA in diabetic rats decreases the cerebral O_2_^−^ generation, apoptosis, and infarction induced by MCAO/Re; all of these effects could be a result of improved antioxidant status in the diabetic brain. Indeed, we observed that AA supplementation to the diabetic rats prevents the diminution of the total AA levels in the cortex associated with ischemia-reperfusion. On the other hand, we found that AA supplementation causes insufficient downregulation of proinflammatory cytokines and has no effect on MPO expression in diabetic rats after MCAO/Re. The relative low dose of AA (100 mg/kg) which we used in this study might not be efficacious against the severe inflammatory responses induced by the combination of stroke and diabetes.

Concentrations of AA in plasma and peripheral extracellular fluid after ingestion are strictly controlled by intestinal absorption, tissue transport, and renal excretion. Chen *et al*. [36] shows that orally administrated AA at doses >200 mg/kg is declined to ≈60 μM in plasma and extracellular fluid of femoral muscle in rats. In the brain, concentration of AA is relatively high compared with that in the plasma [24]. Two mechanisms of AA recruitment into the brain are known: the transport of AA through SVCT2, a specific transporter for AA, and the transport of DHA across the BBB through GLUT1 with subsequent immediate reduction in the brain. The gradation of AA concentrations between the plasma and the CNS is thought to be maintained by SVCT2, because AA is mainly present in the reduced form in the plasma. On the other hand, the cerebroprotective effects of AA supplementation remain controversial, whereas beneficial effects of DHA administration for neuronal oxidative stress and inflammation seem to be well established [37,38,39]. Previous studies do not support acute cerebroprotective effects of treatment with AA under normal conditions because AA is not transported into the brain rapidly, owing to the high affinity and low ability for AA transport by SVCT2. Ahn and colleagues [40] reported that chronic AA supplementation has little or no effect on elevated oxidative stress in STZ-induced diabetic brain, whereas it is efficacious in the liver and kidneys. This phenomenon can be attributed to a lack of SVCT2 expression in endothelial cells of the BBB [41,42]. Recently, the evidence indicating that AA supplementation suppresses diabetes or ischemia-induced oxidative damage in the hippocampus and cortex of experimental animals has been accumulated [43,44,45]. These regions are not only vulnerable to oxidative stress but abundant in SVCT2 compared to other regions in the brain, namely, there may be some kinds of differences in sensitivity for AA supplementation depending on brain regions. Therefore, it seems possible that previous experiments using whole-brain samples might have not been adequate to detect the cerebroprotective effects of AA supplementation.

The system of DHA transport through GLUT1 in the BBB may not substantially contribute to total brain AA concentrations under normal conditions, because DHA concentrations in the plasma are usually only 0%–2% of AA concentrations [19,46]. Nonetheless, during oxidative stress in the CNS such as stroke and/or treatment with DHA, this route could play a significant role in the recruitment of AA [39,47]. Our study demonstrated that diabetes downregulates the expression of GLUT1 in the cortex, in line with previous studies [21,25]. AA supplementation to the diabetic rats markedly upregulated the basal expression of GLUT1; however, it exerted no significant increase in the total AA levels in the cortex of the rats. Taken together, contribution of the DHA transport system through GLUT1 to the cerebroprotection seems to be limited in the diabetic rats. The upregulation of GLUT1 expression by AA supplementation was observed also in the cortex of nondiabetic sham-operated group, despite that there were little changes in the total AA levels in the plasma of the rats. These results may indicate that orally supplemented AA has no direct enhancing effect on the expression of GLUT1 in the nondiabetic rats. However, AA concentration in the plasma is considered to increase within several hours after oral administration and decline to the basal level 24 h after the administration by excretion of the excess of AA, at which we collected the plasma samples from the experimental animals. Therefore, one possibility is that intermittent increases in AA concentration in the plasma repeated for two weeks could stimulate expression of GLUT1 in the cortex. Future research needs to be done with regard to the detailed mechanism underlying the upregulation of GLUT1 expression by AA supplementation.

SVCT2 has been shown to be crucial for maintaining AA for protection against oxidative stress in the CNS. It has been shown that cultured cells lacking this protein are vulnerable to oxidative stress [18]. In the present study, diabetes itself caused no apparent changes in the expression of SVCT2 in the cortex. On the other hand, after MCAO/Re, the expression of SVCT2 in the penumbral cortex, of which the majority seems to be localized in neurons, is upregulated in the nondiabetic rats. Gess and colleagues [20] have demonstrated that SVCT2 is specifically expressed in brain capillary endothelial cells and transports AA across the BBB during the subacute phase after MCAO/Re in mice. We also observed that expression levels of SVCT2 in capillary endothelial cells are increased after MCAO/Re in the nondiabetic rat cortex. This has been proposed as one of the mechanisms that protect the brain against oxidative injury under pathological conditions such as stroke and neurodegeneration. In contrast, we found that the upregulation of SVCT2 expression was abrogated in the diabetic rats, suggesting that the cerebroprotective mechanism via AA recruitment may be disrupted in the diabetic state. The abnormality of endothelial cell function in STZ-diabetic rats has been indicated to be related to the ROS generation in microvascular walls [17]. Seno and coworkers [48] demonstrated that the inflammatory cytokines TNF-α and IL-1β suppress the transport of AA through SVCT2 in human umbilical vein endothelial cells. Indeed, we detected a robust increase in the expression these cytokines in the brain of diabetic rats after MCAO/Re, which may reverse the upregulation of SVCT2 by MCAO/Re itself. AA supplementation to the diabetic rats upregulated the expression of SVCT2 in the cortex and restored the total AA levels to the nondiabetic levels. The antioxidative properties of AA may protect endothelial functions including the transcriptional regulation of SVCT2 against the exacerbated ischemic oxidative injury and the enhanced proinflammatory responses in the diabetic state, and may improve AA transport through SVCT2 in the BBB. Further experiments using radiolabeled tracers or other rodent models such as SVCT2^+/−^ mice are needed to elucidate these cerebroprotective mechanisms of AA, including its effects on the specific transport of AA into the diabetic brain.

## 5. Conclusions

In the present study, we found that chronic AA supplementation inhibits the apoptotic and proinflammatory changes and attenuates the exacerbation of cerebral injury and neurological deficits in the diabetic state. These phenomena could be attributed to the antioxidant activity and anti-inflammatory effects of AA. Diabetes repressed the enhancement of SVCT2 expression induced by ischemia-reperfusion in the neurons and capillary endothelial cells, whereas the downregulated expression of SVCT2 was restored to control levels by AA supplementation. Therefore, chronic AA supplementation may enhance and normalize the transport of AA into the CNS and may thus reinforce the antioxidant defense and alleviate oxidative ischemic injury in the brain of diabetic rats.

## References

[1] Baynes J.W. (1991). Role of oxidative stress in development of complications in diabetes. Diabetes.

[2] Stephens J.W., Khanolkar M.P., Bain S.C. (2009). The biological relevance and measurement of plasma markers of oxidative stress in diabetes and cardiovascular disease. Atherosclerosis.

[3] Biller J., Love B.B. (1994). Diabetes and stroke. Med. Clin. N. Am..

[4] Vinik A., Flemmer M.J. (2002). Diabetes and macrovascular disease. J. Diabetes Compl..

[5] Niizuma K., Endo H., Chan P.H. (2009). Oxidative stress and mitochondrial dysfunction as determinants of ischemic neuronal death and survival. J. Neurochem..

[6] Nakka V.P., Gusain A., Mehta S.L., Raghubir R. (2008). Molecular mechanisms of apoptosis in cerebral ischemia: Multiple neuroprotective opportunities. Mol. Neurobiol..

[7] Niizuma K., Yoshioka H., Chen H., Kim G.S., Jung J.E., Katsu M., Okami N., Chan P.H. (2010). Mitochondrial and apoptotic neuronal death signaling pathways in cerebral ischemia. Biochim. Biophys. Acta.

[8] Warner D.S., Sheng H., Batinić-Haberle I. (2004). Oxidants, antioxidants and the ischemic brain. J. Exp. Biol..

[9] Rolo A.P., Palmeira C.M. (2006). Diabetes and mitochondrial function: Role of hyperglycemia and oxidative stress. Toxicol. Appl. Pharmacol..

[10] Brown G.C., Neher J.J. (2010). Inflammatory neurodegeneration and mechanisms of microglial killing of neurons. Mol. Neurobiol..

[11] Saeed S.A., Shad K.F., Saleem T., Javed F., Khan M.U. (2007). Some newprospects in the understanding of the molecular basis of the pathogenesis of stroke. Exp. Brain Res..

[12] Breckwoldt M.O., Chen J.W., Stangenberg L., Aikawa E., Rodriguez E., Qiu S., Moskowitz M.A., Weissleder R. (2008). Tracking the inflammatory response in stroke *in vivo* by sensing the enzyme myeloperoxidase. Proc. Natl. Acad. Sci. USA.

[13] Jin H.M., Zhou D.C., Gu H.F., Qiao Q.Y., Fu S.K., Liu X.L., Pan Y. (2013). Antioxidant N-acetylcysteine protects pancreatic β-cells against aldosterone-induced oxidative stress and apoptosis in female db/db mice and insulin-producing MIN6 cells. Endocrinology.

[14] Bhattacharya S., Gachhui R., Sil P.C. (2013). Effect of Kombucha, a fermented black tea in attenuating oxidative stress mediated tissue damage in alloxan induced diabetic rats. Food Chem. Toxicol..

[15] Feng B., Yan X.F., Xue J.L., Xu L., Wang H. (2013). The Protective effects of α-lipoic acid on kidneys in type 2 diabetic goto-kakisaki rats via reducing oxidative stress. Int. J. Mol. Sci..

[16] Yamamoto Y., Yamamoto H. (2013). RAGE-mediated inflammation, type 2 diabetes, and diabetic vascular complication. Front. Endocrinol..

[17] Sridulyakul P., Wongeak-In N., Patumraj S. (2012). Correlations between endothelial functions and ROS detection in diabetic microvascular wall: Early and late ascorbic acid supplementation. Int. J. Vasc. Med..

[18] Qiu S., Li L., Weeber E.J., May J.M. (2007). Ascorbate transport by primary cultured neurons and its role in neuronal function and protection against excitotoxicity. J. Neurosci. Res..

[19] May J.M. (2012). Vitamin C transport and its role in the central nervous system. Subcell. Biochem..

[20] Gess B., Sevimli S., Strecker J.K., Young P., Schäbitz W.R. (2011). Sodium-dependent vitamin C transporter 2 (SVCT2) expression and activity in brain capillary endothelial cells after transient ischemia in mice. PLoS One.

[21] Zhang W.W., Zhang L., Hou W.K., Xu Y.X., Xu H., Lou F.C., Zhang Y., Wang Q. (2009). Dynamic expression of glucose transporters 1 and 3 in the brain of diabetic rats with cerebral ischemia reperfusion. Chin. Med. J..

[22] Attele A.S., Zhou Y.P., Xie J.T., Wu J.A., Zhang L., Dey L., Pugh W., Rue P.A., Polonsky K.S., Yuan C.S. (2002). Antidiabetic effects of *Panax ginseng* berry extract and the identification of an effective component. Diabetes.

[23] Takahashi N., Morimoto S., Okigaki M., Seo M., Someya K., Morita T., Matsubara H., Sugiura T., Iwasaka T. (2011). Decreased plasma level of vitamin C in chronic kidney disease: Comparison between diabetic and non-diabetic patients. Nephrol. Dial. Transplant..

[24] Harrison F.E., May J.M. (2009). Vitamin C function in the brain: Vital role of the ascorbate transporter SVCT2. Free Radic. Biol. Med..

[25] Iwata N., Okazaki M., Kamiuchi S., Hibino Y. (2010). Protective effects of oral administrated ascorbic acid against oxidative stress and neuronal damage after cerebral ischemia/reperfusion in diabetic rats. J. Health Sci..

[26] Iwata N., Okazaki M., Kasahara C., Kamiuchi S., Suzuki F., Iizuka H., Hibino Y. (2008). Protective effects of a water-soluble extract from culture medium of *Ganoderma lucidum* mycelia against neuronal damage after cerebral ischemia/reperfusion in diabetic rats. J. Jpn. Soc. Nutr. Food Sci..

[27] Yamazaki Y., Harada S., Tokuyama S. (2012). Post-ischemic hyperglycemia exacerbates the development of cerebral ischemic neuronal damage through the cerebral sodium-glucose transporter. Brain Res..

[28] Muranyi M., Ding C., He Q., Lin Y., Li P.A. (2006). Streptozotocin-induced diabetes causes astrocyte death after ischemia and reperfusion injury. Diabetes.

[29] Li Z., Iwai M., Wu L., Liu H.W., Chen R., Jinno T., Suzuki J., Tsuda M., Gao X.Y., Okumura M. (2004). Fluvastatin enhances the inhibitory effects of a selective AT1 receptor blocker, valsartan, on atherosclerosis. Hypertension.

[30] Faraco G., Fossati S., Bianchi M.E., Patrone M., Pedrazzi M., Sparatore B., Moroni F., Chiarugi A. (2007). High mobility group box 1 protein is released by neural cells upon different stresses and worsens ischemic neurodegeneration *in vitro* and *in vivo*. J. Neurochem..

[31] Caprile T., Salazar K., Astuya A., Cisternas P., Silva-Alvarez C., Montecinos H., Millán C., de Los Angeles García M., Nualart F. (2009). The Na^+^-dependent l-ascorbic acid transporter SVCT2 expressed in brainstem cells, neurons, and neuroblastoma cells is inhibited by flavonoids. J. Neurochem..

[32] Liu K., Mori S., Takahashi H.K., Tomono Y., Wake H., Kanke T., Sato Y., Hiraga N., Adachi N., Yoshino T. (2007). Anti-high mobility group box 1 monoclonal antibody ameliorates brain infarction induced by transient ischemia in rats. FASEB J..

[33] Bradley D.W., Emery G., Maynard J.E. (1973). Vitamin C in plasma: A comparative study of the vitamin stabilized with trichloroacetic acid or metaphosphoric acid and the effects of storage at −70°, −20°, 4°, and 25° on the stabilized vitamin. Clin. Chim. Acta.

[34] Clarke J., Snelling J., Ioannides C., Flatt P.R., Barnett C.R. (1996). Effect of vitamin C supplementation on hepatic cytochrome P450 mixed-function oxidase activity in streptozotocin-diabetic rats. Toxicol. Lett..

[35] Kashiba M., Oka J., Ichikawa R., Kasahara E., Inayama T., Kageyama A., Kageyama H., Osaka T., Umegaki K., Matsumoto A. (2002). Impaired ascorbic acid metabolism in streptozotocin-induced diabetic rats. Free Radic. Biol. Med..

[36] Chen Q., Espey M.G., Sun A.Y., Lee J.H., Krishna M.C., Shacter E., Choyke P.L., Pooput C., Kirk K.L., Buettner G.R. (2007). Ascorbate in pharmacologic concentrations selectively generates ascorbate radical and hydrogen peroxide in extracellular fluid *in vivo*. Proc. Natl. Acad. Sci. USA.

[37] Huang J., Agus D.B., Winfree C.J., Kiss S., Mack W.J., McTaggart R.A., Choudhri T.F., Kim L.J., Mocco J., Pinsky D.J. (2001). Dehydroascorbic acid, a blood-brain barrier transportable form of vitamin C, mediates potent cerebroprotection in experimental stroke. Proc. Natl. Acad. Sci. USA.

[38] Mack W.J., Mocco J., Ducruet A.F., Laufer I., King R.G., Zhang Y., Guo W., Pinsky D.J., Connolly E.S. (2006). A cerebroprotective dose of intravenous citrate/sorbitol-stabilized dehydroascorbic acid is correlated with increased cerebral ascorbic acid and inhibited lipid peroxidation after murine reperfused stroke. Neurosurgery.

[39] Bémeur C., Ste-Marie L., Desjardins P., Vachon L., Butterworth R.F., Hazell A.S., Montgomery J. (2005). Dehydroascorbic acid normalizes several markers of oxidative stress and inflammation in acute hyperglycemic focal cerebral ischemia in the rat. Neurochem. Int..

[40] Ahn T., Yun C.H., Oh D.B. (2006). Tissue-specific effect of ascorbic acid supplementation on the expression of cytochrome P450 2E1 and oxidative stress in streptozotocin-induced diabetic rats. Toxicol. Lett..

[41] García Mde L., Salazar K., Millán C., Rodríguez F., Montecinos H., Caprile T., Silva C., Cortes C., Reinicke K., Vera J.C. (2005). Sodium vitamin C cotransporter SVCT2 is expressed in hypothalamic glial cells. Glia.

[42] Mun G.H., Kim M.J., Lee J.H., Kim H.J., Chung Y.H., Chung Y.B., Kang J.S., Hwang Y.I., Oh S.H., Kim J.G. (2006). Immunohistochemical study of the distribution of sodium-dependent vitamin C transporters in adult rat brain. J. Neurosci. Res..

[43] Bhutada P., Mundhada Y., Bansod K., Tawari S., Patil S., Dixit P., Umathe S., Mundhada D. (2011). Protection of cholinergic and antioxidant system contributes to the effect of berberine ameliorating memory dysfunction in rat model of streptozotocin-induced diabetes. Behav. Brain Res..

[44] Miura S., Ishida-Nakajima W., Ishida A., Kawamura M., Ohmura A., Oguma R., Sato Y., Takahashi T. (2009). Ascorbic acid protects the newborn rat brain from hypoxic-ischemia. Brain Dev..

[45] Jafari Anarkooli I., Sankian M., Vahedi F., Bonakdaran S., Varasteh A.R., Haghir H. (2009). Evaluation of insulin and ascorbic acid effects on expression of Bcl-2 family proteins and caspase-3 activity in hippocampus of STZ-induced diabetic rats. Cell Mol. Neurobiol..

[46] Dhariwal K.R., Hartzell W.O., Levine M. (1991). Ascorbic acid and dehydroascorbic acid measurements in human plasma and serum. Am. J. Clin. Nutr..

[47] Agus D.B., Gambhir S.S., Pardridge W.M., Spielholz C., Baselga J., Vera J.C., Golde D.W. (1997). Vitamin C crosses the blood-brain barrier in the oxidized form through the glucose transporters. J. Clin. Invest..

[48] Seno T., Inoue N., Matsui K., Ejiri J., Hirata K., Kawashima S., Yokoyama M. (2004). Functional expression of sodium-dependent vitamin C transporter 2 in human endothelial cells. J. Vasc. Res..

